# Effect of Dolomite Addition on the Structure and Properties of Multicomponent Amphibolite Glasses

**DOI:** 10.3390/ma15144870

**Published:** 2022-07-13

**Authors:** Adrian Nowak, Malgorzata Lubas, Jaroslaw Jan Jasinski, Magdalena Szumera, Renata Caban, Jozef Iwaszko, Kamila Koza

**Affiliations:** 1 Department of Materials Engineering, Czestochowa University of Technology, Armii Krajowej 19, 42-200 Czestochowa, Poland; adrian.nowak@pcz.pl (A.N.); malgorzata.lubas@pcz.pl (M.L.); renata.caban@pcz.pl (R.C.); jozef.iwaszko@pcz.pl (J.I.); kama130395@interia.pl (K.K.); 2 Materials Research Laboratory, National Centre for Nuclear Research, 05-400 Otwock, Poland; 3 Faculty of Materials Science and Ceramics, AGH University of Science and Technology, Al. Mickiewicza 30, 30-059 Cracow, Poland; mszumera@agh.edu.pl

**Keywords:** structure glass, amphibolite glass, dolomite addition, crystallization, glass-ceramic materials, differential scanning calorimetry DSC

## Abstract

The structure and properties of the glass can be modified by introducing appropriate additives. Dolomite is one of the primary raw materials modifying the properties of glass, in which the essential component is calcium-magnesium double carbonate CaCO_3_∙MgCO_3_. The paper presents the research results on glasses obtained by smelting pure amphibolite and amphibolite modified with 10 and 20% dolomite additives. The raw material used was mined in the Poland region of Lower Silesia. The glass melting process was carried out in an electric furnace at 1450 °C for 2 h. The structure and properties of the glasses and crystallization products were determined by Differential Scanning Calorimetry (DSC), X-ray Diffraction (XRD), Fourier Transform Infrared Spectroscopy (FTIR) and Scanning Electron Microscopy—Energy Dispersive Spectroscopy (SEM-EDS). Viscosity and Vickers microhardness were also measured. It was found that the modification of amphibolite glass by adding dolomite affects the glasses’ properties and structure. The research results determined the effect of dolomite addition on the properties of alumino-silicate glasses in terms of the mineral fibre products used in the construction industry.

## 1. Introduction

Rapid economic and technological development drives the search for new solutions and raw materials in the glass and construction industries. CaO-MgO-Al_2_O_3_-SiO_2_ (CMAS) glasses are among the most essential glass systems with broad applications in these industrial areas due to their excellent chemical and mechanical properties. They are used to produce glass-ceramic materials and glass and mineral fibres [1,2,3,4,5]. These glasses can be obtained from appropriately composed glass sets based mainly on mineral raw materials, e.g., basalt, as in the case of mineral wool production, which is widely used as thermal and acoustic insulation material in heat treatment furnaces, thermal pipelines, construction walls, etc. [6,7,8]. More than 55% of insulating materials work at temperatures up to 200 °C, about 25% in the range 180–400 °C, 5% in the range 400–600 °C, and only 0.1% are installed in conditions where temperature values exceed 600 °C [9,10,11]. The significant disadvantage of mineral fibre wool is the loss of rigidity of the structure. It results, among others, from the usage as a binding agent of synthetic material-phenol-formaldehyde resin with a low range of working temperature (max. 250 °C) [12]. From the construction industry practice, it is known that temperature, moisture and frost can significantly affect the thermal and mechanical properties of insulation materials used in roof panels, facades and foundations [13]. Therefore, producing of a lightweight, non-combustible, weather-resistant, and environmentally friendly insulating material operating at temperatures above 600 °C is a very important subject in the field. Drozdyuk et al. presented the possibility of using bentonite clay as high plasticity binding agent. This material is a layered silicate-montmorillonite group, and its solid mineral particles in suspension contain 63% saponite, 10% quartz, 10% dolomite and other minerals: chlorite, hematite, calcite, apatite, not exceeding 2–3%. Based on the research, the authors showed that the binder makes it possible to produce a thermal insulation material on an industrial scale without changing the production technology of conventional mineral fibre wool. Moreover, the method of applying clay as a binder was successfully implemented in the production line at the Insulation Plant “Zavod Izolatsi” Irpen, Ukraine [14]. The second important factor affecting fibre durability is moisture content. Vrana and Gudmundsson studied the effect of moisture on the properties of cellulose fibres and stone wool over several days [15,16]. They showed that there were slight changes in the water vapour permeability of the fibres. On the other hand, Jerman et al. observed that the thermal conductivity of mineral fibre wool increases rapidly at elevated humidity, from 0.041 W/mK under dry conditions to 0.900 W/mK under saturated conditions [17]. Additionally, Vrana and Bjork studied frost’s effect on different densities of mineral wool. Their results showed that the moisture content was 2.5 or 3 times higher than expected in lower-density insulation materials [18].

In recent years, continuous basalt fibres (CBFs) have also received increasing attention in science and industrial applications of fibre materials [19]. Compared to glass fibre production, the modulus and strength values for high-grade basalt fibres are between E and S2 glass fibres with excellent performance versus cost. Basalt fibres respond to market demand and rising costs in industries that widely use glass fibres e.g., aerospace, automotive and construction [20]. The thermal, chemical, and tribological resistance and insulating properties of basalt fibres can also be a significant added value to the manufacturing of composite materials. The choice of basalt rock as raw material for fibre production is mainly determined by the chemical composition of the fibres, which is similar to that of glass fibres. The optimum SiO_2_ content for CBF production is above 46%. Higher contents improve the fibres’ spinning process and mechanical properties, as does the alumina content [21,22]. The main difference in chemical composition between basalt and glass fibres is the high percentage of iron content in CBF. The proportion of iron oxides Fe_2_O_3_ and FeO influence temperature forming, crystallization ability, and mechanical properties of CBF [23,24,25]. Additional oxides typically present in CBF are CaO, MgO, Na_2_O, K_2_O, and TiO_2_. Bauer et al. investigated basalt fibres’ structure versus property relationships for high-performance applications. They showed that despite using natural basalt rock as raw material, the measured variation in fibre chemistry between batches was less than 1% of the total standard deviation [26]. As mentioned, basalt fibre’s mechanical properties range between E and S2 glass. For the basalt fibres, an inverse tendency of decreasing strength and elongation to break at growing mean fibre diameters is observed and relates to the size effect. In basalt and glass fibres, the mechanical performance increases with higher amounts of silica, alumina, and magnesia (glass formers).

Besides the mentioned basalt, alternative raw materials such as gabbro are also used for insulating materials (mineral wool, CBF) production [27,28]. Therefore, research to find new natural raw materials that meet the criteria for mineral fibre production is still carried out. Considering as interesting raw material for such applications are amphibolites. These are metamorphic rocks whose main components are amphiboles (hornblende, gedrite, cummingtonite) and plagioclase. Besides, the minerals include quartz, epidote, zoisite, garnets, biotite, cordierite, andalusite, sillimanite, tourmalines, titanite, ilmenite, and pyroxenes. Currently, these raw materials are used mainly as broken aggregate in road and railroad construction, as cement additives, or as mineral fertilizer [29,30,31]. However, appropriately composed amphibolite glasses possess suitable properties (crystallization ability, viscosity, acid modulus) to produce glassy insulation materials. This has been studied by Operta and Lampropoulou [32,33]. They showed that the chemical and major mineralogical composition, the heterogeneous characteristics of textures as well as the frequent presence of phyllosilicates due to the weathering of the plutonic studied samples, are expected to contribute to easier grinding and melting of rocks under industrial conditions and to produce new light coloured and competitive stone wool product. Such research confirms that this is a developmental topic. Nevertheless, the high level of industry interest in the selection of raw materials (amphibolites) and parameters for obtaining fibres evidenced mainly by patents results in a lack of scientific publications in this field [34,35]. However, it should be mentioned that amphibolite raw materials often require modification due to their chemical and mineralogical composition. One of the basic raw materials modifying the properties of amphibolite glass is dolomite, whose primary component is a double calcium-magnesium carbonate CaCO_3_∙MgCO_3_ [36]. By introducing dolomite into the glassmaking set, we introduce calcium and magnesium oxides, which influence the thermal stability of glass, its liquidity temperature, and viscosity. Therefore, a properly selected raw material composition, i.e., the proportion of additives and the chemical composition of the set, highly determines the properties and the application of the finished product. In addition, the so-called intrinsic factors, which include viscosity, surface tension, and melt stability, are also important [37,38]. This paper describes the influence of dolomite addition on the structure and properties of amphibolite glasses after melting and directed crystallization in terms of the mineral fibre production used in insulation materials e.g., mineral wool.

## 2. Materials and Methods

**Figure 1:**
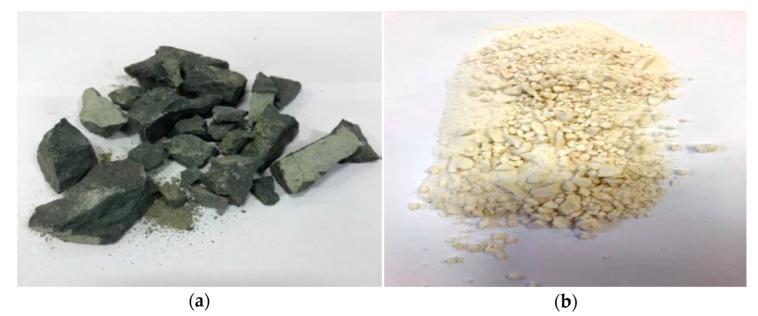
Natural raw materials from the Lower Silesia region of Poland used to melt glasses, (**a**) amphibolite, (**b**) dolomite.

Natural raw materials from the Lower Silesia region of Poland: amphibolite and dolomite, shown in Figure 1, were used in the study. The chemical composition of the raw materials was analyzed by X-ray fluorescence (XRF) spectroscopy using an Axios mAX WDXRF spectrometer with a 4 kW Rh lamp manufactured by PANalytical, Malvern, United Kingdom (Table 1).

Three glassmaking sets were prepared for tests and characterization of the selected raw materials, which contained different proportions of amphibolite and dolomite additions: Set 1*—100 wt.% amphibolite, Set 2*—90 wt.% amphibolite/10 wt.% dolomite, Set 3*—80 wt.% amphibolite/20 wt.% dolomite. The prepared, crumbled, homogenized sets were then placed in alundum crucibles and melted in an electric chamber furnace. The samples were melted at a maximum temperature of 1450 °C for 2 h so that the resulting melt was bubble-free and highly homogeneous. The melt was then cast directly onto a steel plate to ensure a maximum cooling rate. X-ray fluorescence (XRF) spectroscopy determined the chemical composition of the melted samples, as in the case of raw materials. Phase and structural studies were conducted using XRD and FTIR spectroscopic analysis. The X-ray analysis was carried out on a SEIFERT XRD-3003 T-T XRD Eigenmann GmbH, Schnaittach, Germany; X-ray diffractometer with a Co lamp with a wavelength of λ_Co_ = 0.17902 nm, in the range of 2ϴ angles 20–90°. The step size during the scan was 0.1°. IR spectra in the 400–4000 cm^−1^ were obtained with a Fourier spectrometer Bruker Optics-Vertex 70V Billerica, MA, USA. Measurements were made using the powder technique. Absorption of the spectrum was recorded at 128 scans with a resolution of 4 cm^−1^. To characterize critical temperatures of transformations taking place in the obtained samples (T_g_—transformation temperature (glass transition)), T_c_—crystallization temperature, T_m_—melting point), thermal analyses (Dilatometric, DSC) were performed. Dilatometric tests were realized using a Sadamel DA-3 Switzerland dilatometer, while DSC tests were carried out using a NETZSCH DSC-TG STA 449 F3 Jupiter, Selb, Germany; themoanalyzer. The glass samples were heated at the rate of 10 °C/min, in the temperature range of 25 ÷ 1200 °C, in an air atmosphere. The analysis was performed in Al_2_O_3_ crucibles. Moreover, thermal tests allowed to determine stability parameters of the tested glasses according to Hruby (K_H_), Angell (K_A_) and Saad-Poulain (K_SP_) equations (Equations (1)–(3)) [39,40,41]. The process of directed crystallization of the melted amphibolite glasses was carried out at the following parameters: time 5 and 10 h, temperature 800 °C, 900 °C and 1000 °C. In addition, to determine the changes taking place as a consequence of the directed crystallization process on the structure and properties of amphibolite glasses, XRD and SEM-EDS examinations using a JOEL JSM-6610LV, Peabody, MA, USA; scanning microscope were performed. Microhardness tests were performed both for the as-received glasses and glasses after heat treatment. The Vickers microhardness values were measured using a SHIMADZU, Kioto, Japan; microhardness tester under a load of 50 g. The dwell time of 10 s was constant for all the samples. Microhardness values were calculated using the following equation: HV = A (p/d2) kg/mm^2^, where A is a constant equal to 1854.5, p is the applied load (g), and d is the average diagonal length (μm); and converted from kg/mm^2^ to MPa. Viscosity was determined for all tested glasses using the one-point method, where the base for viscosity curve determination is the T_g_ temperature received from the DSC analysis. The viscosity of the tested glasses was then determined using the relationship initially presented in Formula (4) [42].

## 3. Results and Discussion

### 3.1. Study of Amphibolite Glasses after the Melting Process

**Figure 2:**
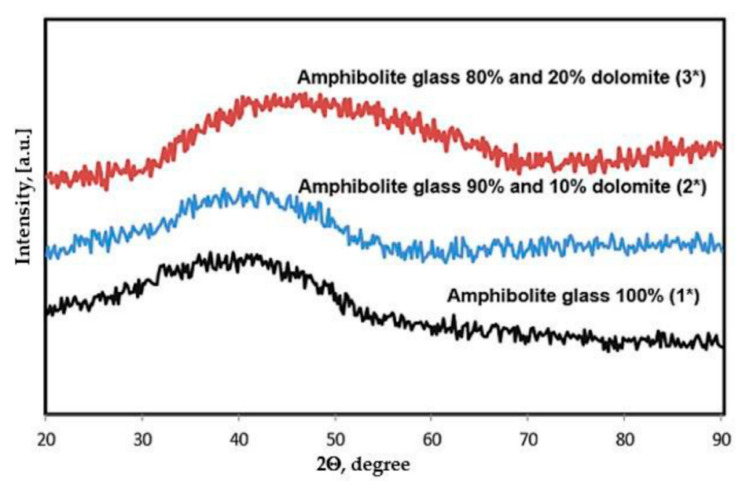
X-ray diffractograms of amphibolite glasses after the melting process.

In the first stage of the research, after the melting process of the prepared glass sets, XRD phase analysis was performed, and the chemical composition was determined using the XRF method. The analyses performed are presented in Figure 2 and Table 2. The diffractograms showed only an increased background in the range of 30–50°, characteristic of amorphous materials, which confirms that an amorphous material-glass—was obtained in the melting stage [43].

**Figure 3:**
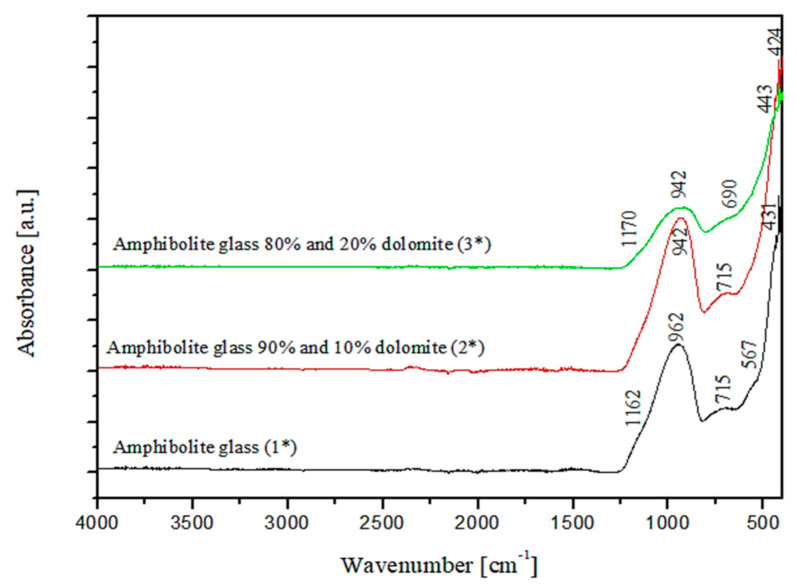
FTIR spectra of amphibolite glasses after the melting process.

The chemical composition analysis of the obtained glasses confirmed that they are multicomponent aluminosilicate glasses of the SiO_2_-Al_2_O_3_-MgO-CaO system. As can be seen from the literature data, research on this type of glasses focused, among others, on their application to the production of glass-crystalline materials, very often obtained based on waste materials from the construction industry [44,45]. The results of structural studies with FTIR spectroscopy of the produced amphibolite glasses with different dolomite contents are presented in Figure 3.

**Figure 4:**
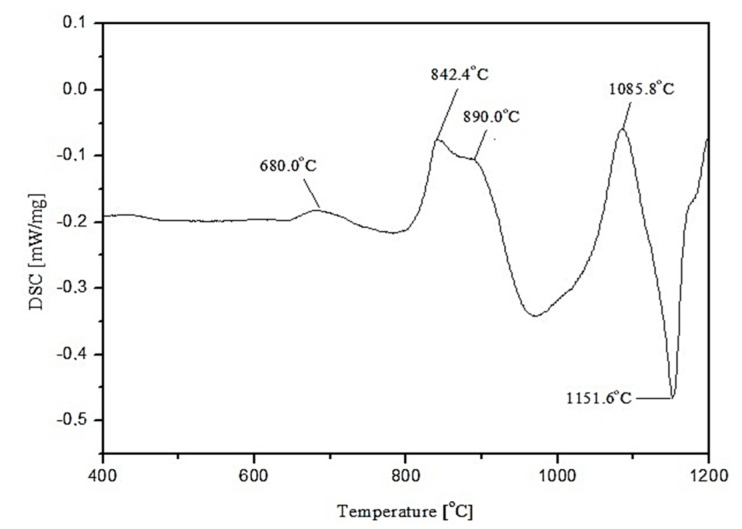
DSC curve of 100 wt.% amphibolite glass (Set 1*).

**Figure 5:**
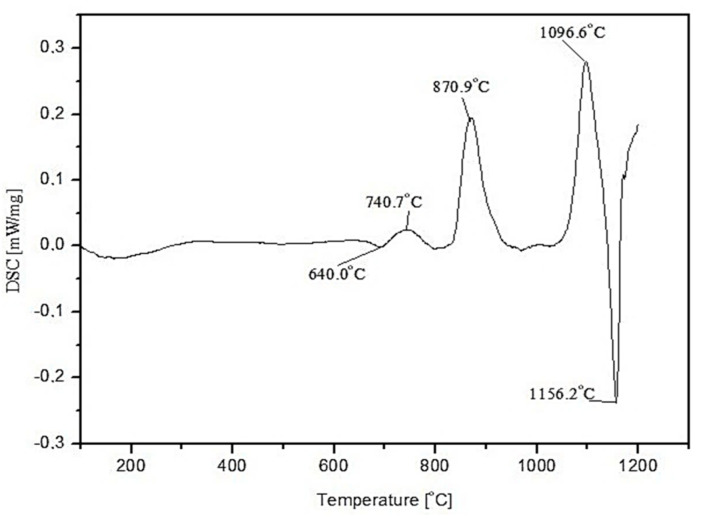
DSC curve of amphibolite glass with 10 wt.% dolomite addition (Set 2*).

**Figure 6:**
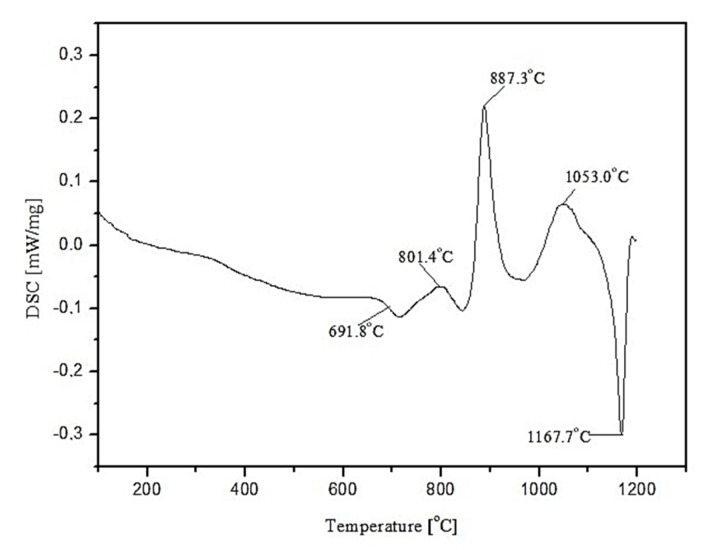
DSC curve of amphibolite glass with 20 wt.% dolomite addition (Set 3*).

**Figure 7:**
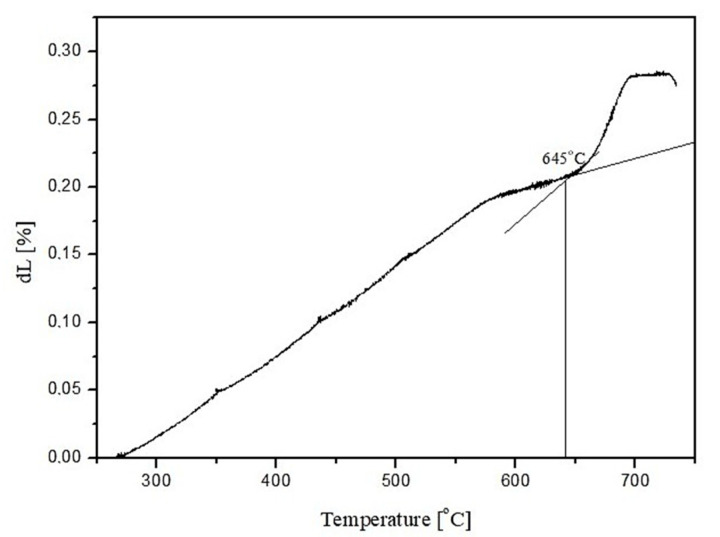
Dilatometric curve of 100 wt.% amphibolite glass (Set 1*).

Three main absorption bands are visible in the FTIR spectra of the studied amphibolite glasses. Peaks are located at approx. 962 (942), 715 (690) and 431 (443/424) cm^−1^. The most intense bands, located in the range 1400–800 cm^−1^ are attributed to asymmetric stretching vibrations of two bond types: Si-O-(Si), and (Al) [46]. Much less intense complex bands in the range of 800–650 cm^−1^ correspond to symmetric bending vibrations of Si-O-Si and Si-O-Al [47]. The band located in the range of 550–400 cm^−1^ can be related to bending vibrations originating from O-Si-O and O-Al-O [48,49]. The first group is the most sensitive to changes in a lattice structure and usually represents a superposition of several bands located close to each other [50]. Based on the obtained spectra, it was observed that adding dolomite and introducing CaO and MgO oxides at the expense of SiO_2_, causes the breaking of some of the Si-O-Si and Si-O-Al bonds. The network depolymerizes under the influence of modifiers, which is particularly evident in the case of glass modified with 20 wt.% dolomite addition, where a significant decrease in half-width of bands in the wavenumber range 1400–800 cm^−1^ is visible and a shift of all bands towards lower wavenumber values. In the next step of the research, transformation temperatures (T_g_, T_c_, T_m_) of the glasses were determined by DSC thermal analysis as shown in Figure 4, Figure 5 and Figure 6. In the case of the 100% amphibolite (Set 1*) sample, the transformation temperature could not be accurately determined from the DSC curve, so additional dilatometric tests were carried out finally to determine T_g_ (Figure 7).

The thermal analysis results were used to determine the basic parameters of the thermal stability of the glasses according to Hruby (K_H_), Angell (K_A_) and Saad-Poulain (K_SP_) using the following equations [39,40]:

(1) $$K_{\begin{array}{c} H \end{array}}=\frac{T_{c}-T_{g}}{T_{m}-T_{c}}$$

(2) $$K_{A\;}={\;T}_{c}-T_{g}$$

(3) $$K_{\mathrm{SP}\;}=\frac{(T_{c}-{\;T}_{g})\;\left(T_{\mathrm{cmax}}^{}-{\;T}_{c\;}\right)}{T_{g}\;}$$

where: T_g_—glass transition temperature, T_c_—crystallization start temperature, T_cmax_—crystallization maximum temperature, T_m_—melting point.

The Angell parameter (K_A_) is a simple relationship; it only considers the difference between crystallization and glass transition temperatures. On the other hand, according to Hruby and Saad-Poulaine, the relationships are more complex. They take into account other correlations affecting the propensity to vitrification and consider the three most essential transformation temperatures during glass heating (T_g_, T_c_, T_m_). Table 3 summarizes the DSC results together with the glass stability parameters.

The thermal analysis results showed that modifying the amphibolite glasses with dolomite changes their characteristic temperatures and thus affects thermal stability. DSC curves showed that the glass transition temperature (T_g_) of the obtained amphibolite glasses is between 640–690 °C. Exothermic processes related to glass crystallization were visible at higher temperatures, which depends mainly on the chemical composition. Additionally, the multiphase crystallization process was recorded for all the amphibolite glasses. The exothermic crystallization effect was the weakest and broad for the unmodified amphibolite glass (Set 1*), thus, the most intensive crystallization effect was observed for glass modified with 20 wt.% dolomite addition (Set 3*). For the studied glasses, it is also characteristic that an endothermic effect is visible after the exothermic effect related to the complete or partial melting of the crystalline phases. In general, the applied modification of glasses with dolomite had a positive effect on the material’s thermal stability. The addition of dolomite in the amount of 10 wt.% (Set 2*) caused an increase in the values of K_A_ and K_H_, K_SP_ parameters in comparison to pure amphibolite glass (Set 1*). The higher values of these parameters indicate a decrease in the glass’s ability to crystallize, which is beneficial, e.g., in mineral fibre production. On the other hand, an increase in the proportion of dolomite to 20 wt.% increases the glass transition temperature from 640 °C (Set 1*) to 692 °C (Set 3*), with a simultaneous decrease in glass stability. It is known that MgO in the glass structure can take both the coordination numbers 6 and 4 [51,52]. This means that it can act as a modifying ion (LK 6) similarly to CaO, or build into the network as a bond-forming ion (LK4). In the case of glasses containing in their chemical composition a high proportion of metal ions that can occur in different coordination numbers, any increase in the amount of free oxygen in the glass mass favours the adoption of lower coordination by these ions. The increase of metal ions in coordination number 4 results in the incorporation of these ions into the glass bond, thus strengthening it, which contributes to an increase in the stability of glass with 20% dolomite addition [53]. According to literature data, the higher K_H_ value for glass, the higher its resistance to crystallization during heating and ability to vitrify during cooling [54,55]. A lowering of the K_SP_, K_A_, and K_H_ values implies a higher ability to crystallize, which is particularly important during the lifetime of the glasses, depending on their intended use. However, in the case of mineral fibres, this can contribute to crushing and loss of the required strength properties [56].

### 3.2. Study of Amphibolite Glasses after the Crystallization Process

**Figure 8:**
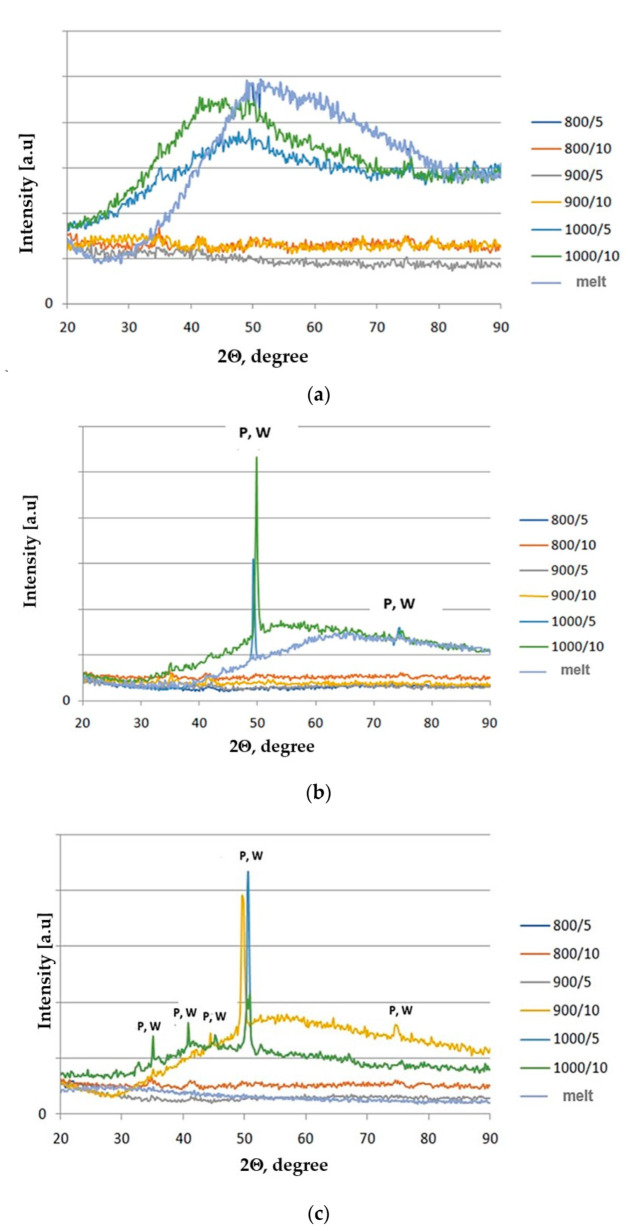
X-ray diffractograms of amphibolite glasses after after crystallization process: (**a**) 100 wt.% amphibolite (Set 1*), (**b**) 90 wt.% amphibolite + 10 wt.% dolomite (Set 2*), (**c**) 80 wt.% amphibolite + 20 wt.% dolomite (Set 3*); P—Pyroxene, W—Wollastonite.

**Figure 9:**
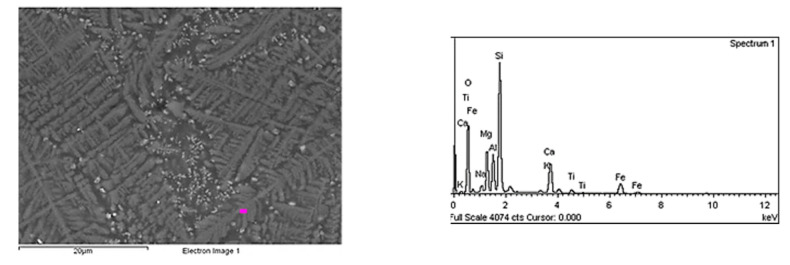
SEM-EDS image and analysis of amphibolite glass modified with 20 wt.% dolomite addition (Set 3*) after crystallization at 1000 °C for 10 h.

**Figure 10:**
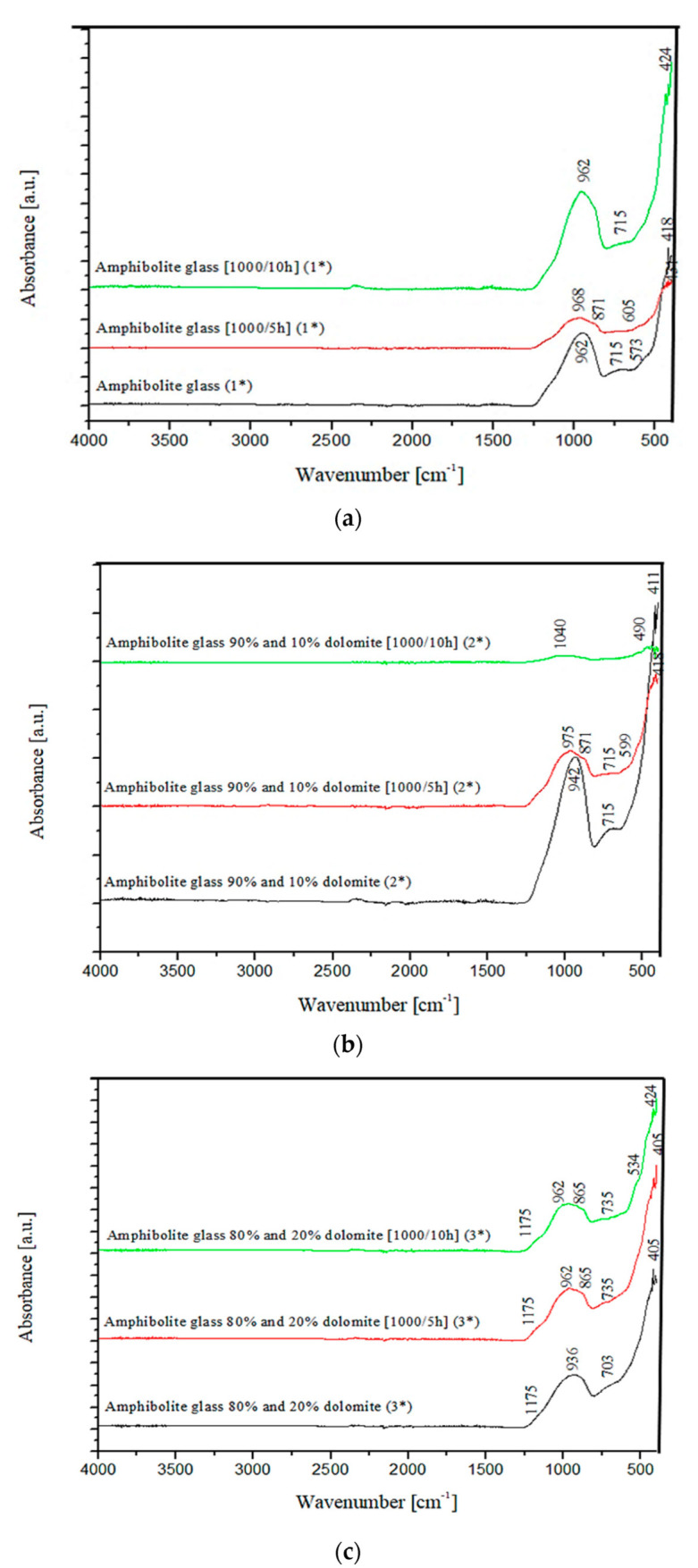
FTIR of amphibolite glasses selected before and after crystallization process: (**a**) 100 wt.% amphibolite (1*), (**b**) 90 wt.% amphibolite + 10 wt.% dolomite (2*), (**c**) 80 wt.% amphibolite + 20 wt.% dolomite (3*).

The DSC results shown in Table 3 were used to determine the parameters of the directed crystallization process, which were ultimately carried out for temperatures of 800 °C, 900 °C and 1000 °C and times 5 and 10 h. The structural investigation results using XRD and FTIR spectroscopic analysis for the crystallized glasses are shown in Figure 8, Figure 9 and Figure 10.

The XRD results confirmed the amorphous character only for a glass containing 100 wt.% amphibolite both after the melting and crystallization processes. This is evidenced by the visible raised, extended background at 34–50° (Figure 8a–c). In the glasses modified with dolomite after the crystallization process, apart from the increased background, peaks characteristic of amorphous materials were observed, confirming the presence of crystalline phases. Based on the analysis, it was found that the phases are represented mainly by pyroxenes (diopside (Ca(Mg,Al)(Si,Al)_2_O_6_; JCPDS no. 41-1370) and wollastonite (CaSiO_3_, JCPDS no. 043-1460). The main crystalline phase is pyroxenes (augite, diopside). The addition of dolomite introduced additional Ca^+^ and Mg^+^ ions into the glasses, contributing to the crystallization of pyroxene phases. Pyroxenes are minerals with various chemical compositions. A wide variety of ionic and isomorphic substitutions of multiple elements occurs among the group of pyroxenes. Some of the Al^3+^ ions from the base glass can enter the pyroxene structures, replacing the Mg^2+^ and Si^4+^ [57]. The Ca^2+^ ion is one of the major ions, and its effective radius is close to Pb^2+^ and Ba^2+^, which exhibits minimal mobility in the glass lattice. At the same time, Mg^2+^ has a smaller size and greater mobility. Therefore, the rate of diffusion of Ca^2+^ ions mainly determines the formation of diopside [58]. In the case of the glasses modified with the 10 wt.% addition of dolomite, peaks from the crystalline phases appear only at 1000 °C for the glasses heated for 5 and 10 h. For the glasses with the 20 wt.% addition of dolomite, peaks appear at 900 °C after 10 h of heating, indicating crystalline phase formation. These results confirm that the addition of dolomite increases the ability of amphibolite glasses to crystallize. However, a higher dolomite content reduces the thermal stability of the glasses. Figure 9 shows the microstructure of the amphibolite glass obtained from Set 3*, after the crystallization process (1000 °C/10 h). The obtained SEM-EDS results confirmed the XRD studies that pyroxenes are the main crystallizing phase in the examined multicomponent amphibolite glasses.

After the directed crystallization process, the amphibolite glasses’ spectroscopic studies with FTIR showed slight changes (Figure 10). In addition to the three main bands discussed in the spectra of the base glass (Figure 3), broadening of the bands in the range of 1400–850 cm^−1^ and a division into two bands at values of approx. 962 and approx. 865 cm^−1^ was observed. This proves the formation of crystalline phases in the crystallization process of the glasses—mainly pyroxenes—in the diopside band at 865 cm^−1^ [59,60]. The bands in the range 800–650 cm^−1^ for glass after directed crystallization (1000 °C/10 h) show spectra with a hardly visible significant half-width. These changes confirm the depolymerization of the glass lattice, a loss of Si-O-Si, Si-O-Al [61].

The results of the DSC thermal analysis were also used to determine the viscosity of the multicomponent amphibolite glasses (Table 4), a parameter that is very important from the point of view of glass fibre production. The one-point method was used to determine the parameter, where the transformation temperature (Tg) is used to determine the viscosity of the glass, according to the relation [42]:

(4) $$\log\eta \;=13.4-C_{1\;}+\frac{C_{1}C_{2}}{T-\mathrm{Tg}+C_{2}}$$

where: T—test temperature [°C], T_g_—transformation temperature (determined for the tested glasses) C_1_, C_2_—constant.

The dependence of the glass viscosity on the chemical composition allows two areas of viscosity to be distinguished: low-temperature η > 10^6^ dPas, affecting the crystallization of the glasses, and high-temperature, within the limits of η < 10^6^ dPas, important from the point of view of the melting and fibre formation process. Dolomite addition to the amphibolite glass in the amount of 10 wt.% slightly reduces the viscosity of the glass. On the other hand, increasing the amount of dolomite to 20 wt.% causes a slight increase in viscosity in the entire temperature range. In the investigated glasses, the sum of the oxides of alkali metals and alkaline earth metals is greater than the alumina content. Ratio [R_2_O+RO]/[Al_2_O_3_] > 1 (Table 2). It is known that Al_2_O_3_ can be a glass-forming component [62], and when Al^3+^ ions enter the [AlO_4_] structure, there should be enough alkali metal and alkaline earth metal cations to maintain the electric charge balance. Based on literature data, it is known that Ca^2+^ ions take precedence over Mg^2+^ ions in charge of the compensation of Al^3+^ ions and lead to the formation of more stable Ca[AlO_4_]^5−^ tetrahedral structures, which in turn results in increased viscosity > 10^6^ dPas, important from the point of view of the melting and fibre formation process [63,64].

The results of microhardness tests of amphibolite glasses by the Vickers method (Table 5) showed that the microhardness of amphibolite glasses increases with dolomite content. Moreover raising the MgO and CaO content at the expense of SiO_2_ also improves the microhardness of the glasses [65,66] same as the increasing proportion of crystalline phases in the glass matrix for crystalline glasses [67]. This is particularly evident in the case of glasses after the crystallization process modified with 20 wt.% dolomite addition (Set 3*).

## 4. Conclusions

Based on the results, it was found that the modification of amphibolite glasses with dolomite in various proportions affects their structure and properties. The structure of the glasses studied changed depending on the amount of dolomite in the glass and the conditions of the crystallization process carried out. The glass stability coefficients determined from the results of DSC thermal analysis showed that at a dolomite content of 10 wt.% the glass stability is higher than that of pure amphibolite glass (Set 1*). In addition, dolomite reduces viscosity, which is very beneficial in the context of mineral fibre production. The crystallization process carried out showed that the crystalline phases in the studied glasses are mainly represented by pyroxenes-diopside and wollastonite. In the case of the addition of 10 wt.% of dolomite, the crystalline phases appear only at a temperature of 1000 °C, whereas when the dolomite content is 20 wt.%, the glass crystallizes already at a temperature of 900 °C. Moreover, when analyzing microhardness results, it was found that the addition of dolomite and optimization of the crystallization process increases the strength of amphibolite glasses, which is very important for the production of mineral fibres for many industrial sectors. The authors conduct further research on amphibolite glasses focusing on applications in the construction industry, including insulation materials, as well as reinforcement of composites, and building materials for structural applications.

## Figures and Tables

**Table 1:** Chemical composition analysis of raw materials used to melt glasses [wt.%].

Raw Material	Composition, [wt.%]												
	Na_2_O	MgO	Al_2_O_3_	SiO_2_	P_2_O_5_	SO_3_	K_2_O	CaO	TiO_2_	MnO	Fe_2_O_3_	ZrO_2_	PbO
Amphibolite	5.41	4.78	14.1	54.6	<0.10	<0.1	0.22	6.08	1.08	0.25	10.3	<0.1	-
Dolomite	0.37	36.93	1.37	3.27	0.06	0.08	0.19	59.34	0.04	0.19	0.83	0.26	0.04

**Table 2:** Chemical composition of amphibolite glasses after the melting process [wt.%].

Glass	Component, [wt.%]											
	SiO_2_	Al_2_O_3_	CaO	MgO	Fe_2_O_3_	K_2_O	Na_2_O	TiO_2_	P_2_O_5_	MnO	ZrO_2_	SO_3_
Set 1* Amphibolite Glass	54.6	14.1	6.08	4.78	10.3	0.22	5.41	1.08	0.10	0.25	0.10	0.10
Set 2* Amphibolite Glass 90 wt.%/Dolomite 10 wt.%	49.47	12.83	11.41	8.00	9.35	0.22	4.91	0.98	0.09	0.24	0.11	0.10
Set 3* Amphibolite Glass 80 wt.%/Dolomite 20 wt.%	44.33	11.55	16.73	11.21	8.41	0.21	4.40	0.87	0.09	0.24	0.12	0.09

**Table 3:** Characteristic temperatures of studied amphibolite glasses determined by DSC thermal analysis.

Glass	T_g_ [°C]	T_c1_ [°C]	T_c1max_ [°C]	T_c2max_ [°C]	T_c3max_ [°C]	T_m_ [°C]	K_A_	K_H_	K_SP_
Set 1* 100 wt.% Amphibolite	645	665	681	842	1086	1151	20	0.04	0.50
Set 2* 90 wt.% Amphibolite/10 wt.% Dolomite	640	675	741	871	1097	1156	35	0.07	3.61
Set 3* 80 wt.% Amphibolite/20 wt.% Dolomite	692	710	801	887	1050	1168	18	0.04	2.37

**Table 4:** Viscosity coefficients of glasses determined by the one-point method.

Glass	Temperature, °C											
	660	700	740	860	900	940	980	1020	1060	1100	1140	1180
	logη, dPas											
Amphibolite glass T_g_ = 640 °C	13.6	11.4	9.8	6.5	5.8	5.2	4.6	4.2	3.8	3.4	3.2	2.8
Amphibolite glass 90 wt.%/10 wt.% Dolomite, T_g_ = 645 °C	13.0	11.0	9.4	6.3	5.6	5.0	4.5	4.1	3.7	3.3	3.0	2.7
Amphibolite glass 80 wt.% /20 wt.% Dolomite, T_g_ = 692 °C	14.3	12.0	10.2	6.8	6.0	5.3	4.8	4.3	3.9	3.5	3.3	2.9

**Table 5:** Vickers microhardness results for amphibolite glasses after melting and the crystallization process.

Melt/Crystallization Parameters [°C]/Time h	Type of Glass		
	Set 1 Amphibolite Glass 100 wt.%	Set 2 Amphibolite Glass 90 wt.% Dolomite 10 wt.%	Set 3 Amphibolite Glass 80 wt.% Dolomite 20 wt.%
	HV 0.05	HV 0.05	HV 0.05
Melt (glass)	690	751	769
800/5	693	756	773
800/10	695	712	757
900/5	711	759	805
900/10	773	840	871
1000/5	736	813	835
1000/10	745	807	855

## Data Availability

The data presented in this study are available on request from the corresponding author. The data are not publicly available due to the possibility of use in further research.
